# Editorial: Global excellence in molecular immunology and therapeutics: Africa 2021

**DOI:** 10.3389/fimmu.2023.1247148

**Published:** 2023-07-12

**Authors:** Tarek A. Ahmad, Yusuf O. Omosun

**Affiliations:** ^1^ Library Sector, Bibliotheca Alexandrina, Alexandria, Egypt; ^2^ Microbiology, Biochemistry and Immunology Department, Morehouse School of Medicine, Atlanta, GA, United States

**Keywords:** molecular immunology, Africa, tuberculosis, COVID, malaria, cancer

Africa is the only continent that stretches from above the Tropic of Cancer to below the Tropic of Capricorn. Regardless of the misrepresentation of Africa in the present map of the world, Africa is the second largest and most populated continent after Asia and has plenty of natural resources, which have not been used judiciously for a number of reasons. These together make Africa a continent of contradictions between hot tropical and freezing subarctic, dry arid areas and rainy jungles, and big lakes and water scarcity, not to mention the largest number of megafauna and range of plant biodiversity on the one hand and famine and pollution on the other. The continent has high disease incidence and pollution while having very high population growth. However, Africa is the continent with the smallest GDP on earth. Considering all these conditions, Africa still has a share of the world’s scientific research, through its national institutions or African scientists in research groups abroad. In both cases, these scientists aim to investigate diseases that are not necessarily priorities to Western sponsors but cause serious health burdens to Africans.

The African environment and hygiene level have a role to play in determining diseases of significance in Africa. Overall, 70% of people living with HIV can be found in Africa, and 85% of people on the continent are affected by malaria, while tuberculosis and leprosy are also a major burden. In addition, there is high mortality in children infected with pneumococci, enterococci, and rotavirus infections. Parasitic diseases such as Leishmania, Trypanosoma, Filaria, Schistosoma, and several other diseases such as rabies and dengue fever still affect a considerable number of Africans. All these diseases, together with emerging diseases such as Ebola, Marburg, Lassa, and COVID-19, have had a considerable effect on the quality of life of the average African.

Frontiers in Immunology put forward a Research Topic for African scientists to showcase publications that display scientific achievements in Africa in the field of molecular immunology centered on working on the diseases scourging the continent. These included understanding the molecular mechanisms of disease pathogenesis, vaccinology, therapeutics, and diagnostic tools. In this Research Topic, there were seven insightful studies addressing the molecular mechanisms of host–pathogen interactions in emerging pandemic pathogens such as COVID-19, persistent pathogens such as malaria and TB, and cancer pathogenesis.

As a part of the international movement to combat the COVID-19 pandemic, the Egyptian group of Behairy et al. evaluated the effect of the single-nucleotide polymorphism (SNP) change in the gene encoding the innate human β-defensins type 2 (*h*BD-2) on susceptibility to COVID-19. Their bioinformatic investigations confirmed that SNP changes could alter the innate immunity to the virus, and this can manifest as varied susceptibility to infection and also determine the degree of severity between individuals. The finding paves the way for further experimental and clinical evaluation for procedures that can be applied to manage and diagnose the pathogen. Simultaneously, the Egyptian group of Yasseen et al. studied the non-conventional role of platelets in immunity. They investigated the molecular mechanisms of aggregation and activation of the leukocyte–platelets complex associated with the clinical severity and mortality outcomes of COVID-19 patients. They revealed that the morphological and functional changes in hyperactive platelets during infection can be used as a very accurate predictor of mortality associated with COVID-19.

The global accelerated momentum to produce prophylactic vaccines against pathogens causing pandemics such as COVID-19 highlighted the global inequality of vaccine availability for several African countries due to the lack of local infrastructure. The WHO, in association with other partners, has launched the mRNA technology transfer program for the use of mRNA technology to produce COVID-19 or other vaccines. The group of Kairuz et al., from the University of Witwatersrand, highlighted the recent advances in the field of synthetic mRNA therapies and vaccines. This technology may be applied further to produce necessary neoantigen vaccines, therapeutic proteins, gene editing for genetic disorders, and vaccine development against HIV, TB, and malaria.

A lot of effort has been made in studying malaria in Africa. The group of Thiam et al., from the University of Ghana and Institut Pasteur of Dakar, Senegal, screened for some phenotypic variation in the malaria parasite and confirmed that there was low level of diversity across Western Africa. This approach was followed as a step to decipher the conserved epitopes found on malaria merozoites that can be used as vaccine building blocks. A multinational group led by Azam et al., including collaborators from Africa, proved the applicability of using CRP measurement to assess the treatment efficiency for TB since using the molecular bacterial load assay based on 16S-RNA evaluation is not affordable for several health systems in Africa.

The incidence of cancer is on the rise on the African continent. In Casablanca, the Moroccan group of Ghazi et al. reviewed research proposing the use of chimeric antigen receptor T-cells (CART) therapy for colorectal solid tumors. The researchers supported the application of CART therapy for colorectal cancer while highlighting that clinical trials are necessary to provide insights into the spectrum of patients who can benefit from this treatment. The Ethiopian group of Shibabaw et al. reviewed the use of molecules that can downregulate the production of IL-17A, a major stimulant for metastatic breast cancer. The targeting of IL-17A signaling pathways provides a promising approach for novel treatments.

Overall, we live in a world with extensive exchange of capacity at all levels. Pathogens spread between continents and threaten the lives of human beings worldwide. The lesson we learned from the COVID-19 pandemic is that exchanging data and cooperating in the production of lifesaving products are essential to protect humanity. Having a will to build centers of excellence in Africa with African human capacity and infrastructure will support the efforts of Africans in tracking, studying, and limiting the spread of pathogens, as well as creating skilled regional employment opportunities in the sciences.

## Author contributions

TA drafted the editorial. YO revised and approved the manuscript.

